# MVA vector expression of SARS-CoV-2 spike protein and protection of adult Syrian hamsters against SARS-CoV-2 challenge

**DOI:** 10.1038/s41541-021-00410-8

**Published:** 2021-12-03

**Authors:** Clement A. Meseda, Charles B. Stauft, Prabhuanand Selvaraj, Christopher Z. Lien, Cyntia Pedro, Ivette A. Nuñez, Amy M. Woerner, Tony T. Wang, Jerry P. Weir

**Affiliations:** Division of Viral Products, Center for Biologics Evaluation and Research, Food and Drug Administration, 10903 New Hampshire Ave, Silver Spring, MD 20993 USA

**Keywords:** Microbiology, Vaccines

## Abstract

Numerous vaccine candidates against SARS-CoV-2, the causative agent of the COVID-19 pandemic, are under development. The majority of vaccine candidates to date are designed to induce immune responses against the viral spike (S) protein, although different forms of S antigen have been incorporated. To evaluate the yield and immunogenicity of different forms of S, we constructed modified vaccinia virus Ankara (MVA) vectors expressing full-length S (MVA-S), the RBD, and soluble S ectodomain and tested their immunogenicity in dose-ranging studies in mice. All three MVA vectors induced spike-specific immunoglobulin G after one subcutaneous immunization and serum titers were boosted following a second immunization. The MVA-S and MVA-ssM elicited the strongest neutralizing antibody responses. In assessing protective efficacy, MVA-S-immunized adult Syrian hamsters were challenged with SARS-CoV-2 (USA/WA1/2020). MVA-S-vaccinated hamsters exhibited less severe manifestations of atypical pneumocyte hyperplasia, hemorrhage, vasculitis, and especially consolidation, compared to control animals. They also displayed significant reductions in gross pathology scores and weight loss, and a moderate reduction in virus shedding was observed post challenge in nasal washes. There was evidence of reduced viral replication by in situ hybridization, although the reduction in viral RNA levels in lungs and nasal turbinates did not reach significance. Taken together, the data indicate that immunization with two doses of an MVA vector expressing SARS-CoV-2 S provides protection against a stringent SARS-CoV-2 challenge of adult Syrian hamsters, reaffirm the utility of this animal model for evaluating candidate SARS-CoV-2 vaccines, and demonstrate the value of an MVA platform in facilitating vaccine development against SARS-CoV-2.

## Introduction

A large number of COVID-19 vaccines are currently in development, many of which are in advanced clinical trials. Candidate vaccines include a variety of platforms such as inactivated virus vaccines, nucleic acid vaccines, protein subunit vaccines, viral vector vaccines, and live attenuated vaccines (see https://www.who.int/publications/m/item/draft-landscape-of-covid-19-candidate-vaccines for an updated list of candidate vaccines). The majority of these vaccine candidates are designed to elicit a protective immune response to the SARS-CoV-2 spike (S) protein, the major structural protein of the virus that has an essential role in the attachment and infection of host cells^1^. Modified vaccinia virus Ankara (MVA) vectors have shown promise as potential candidate vaccines for numerous pathogens, including related coronaviruses such as SARS-CoV-1 and MERS^2,3^. The advantages of an MVA vector platform as a vaccine have been described previously and benefits include the ability to elicit humoral and cellular immune responses to expressed heterologous genes and demonstrated safety features including restricted replication in mammalian cells^4,5^.

While SARS-CoV-2 S is the most common antigen expressed by candidate COVID-19 vaccines, several forms of S have been incorporated into vaccine candidates including the full-length S expressed by the SARS-CoV-2 virus, stabilized prefusion forms of S designated as 2P or 6P, and fragments of S such as the receptor-binding domain or the N-terminal domain. Here, we describe the construction of MVA vectors expressing several versions of SARS-CoV-2 S including the full-length unmodified S protein (i.e., no stabilizing or other amino acid modifications), the receptor-binding domain, and a soluble S ectodomain. Immunogenicity was evaluated in dose-ranging studies in mice. To further evaluate the immunogenicity and to assess protective efficacy, the MVA vector expressing full-length unmodified S (MVA-S) was used to immunize adult Syrian hamsters that were subsequently challenged with SARS-CoV-2. In this hamster challenge model, pathology was prevented in animals vaccinated with MVA-S, as evident in significant reductions in gross pathology scores and weight loss. MVA-S-vaccinated hamsters exhibited less severe or nonexistent manifestations of atypical pneumocyte hyperplasia, hemorrhage, vasculitis, and especially consolidation, compared to control animals. The results demonstrate the utility of the MVA platform in evaluating candidate antigens to facilitate vaccine development against SARS-CoV-2.

## Results

### Construction of recombinant MVA vectors expressing SARS-CoV-2 antigens

**Fig. 1:**
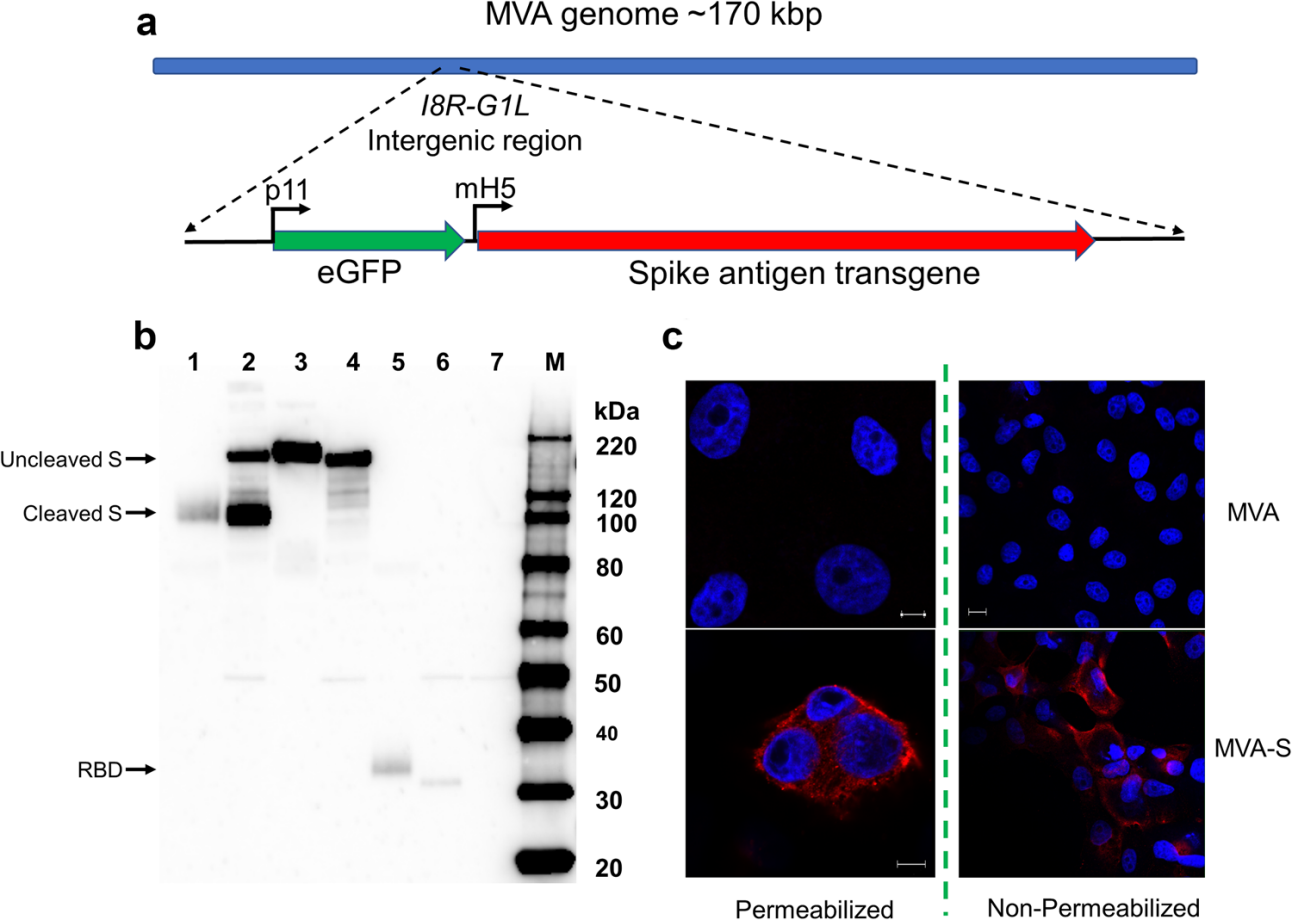
Construction and characterization of recombinant MVA SARS-CoV-2 antigens. **a** Schematic diagram of the MVA vector design. **b** Western blot analysis. Confluent monolayers of Vero cells in a 6-well plate were infected with MVA-S, MVA-ssM, MVA-RBD, or MVA. The supernatants and the infected cells were collected after 6 h and processed for Western blot with a rabbit anti-SARS-CoV-2 spike protein polyclonal antibody. Images were captured using an LAS3000 imaging system (Fujifilm). Lane 1: MVA-S supernatant, lane 2: MVA-S cell lysate, lane 3: MVA-ssM supernatant, lane 4: MVA-ssM cell lysate, lane 5: MVA-RBD supernatant, lane 6: MVA-RBD cell lysate, lane 7: MVA cell lysate, lane M: MagicMark protein molecular weight markers. The blot was derived from a single experiment that was repeated one time. The uncropped blot is shown in Supplementary Fig. 1. **c** Confocal microscopy. MVA- or MVA-S-infected Vero E6 cells were fixed in 4% paraformaldehyde. Cells were either permeabilized in 0.2% Triton X-100 or left untreated (non-permeabilized) and subsequently stained with a polyclonal anti-SARS-CoV-2 spike antibody (GTX135356) (red). Nuclei were counterstained with DAPI (blue). Scale bars represent 5 µm (left) and 10 µm (right).

Recombinant MVA vectors were designed and constructed to express full-length SARS-CoV-2 spike protein (MVA-S), a soluble spike ectodomain (MVA-ssM), or the SARS-CoV-2 receptor-binding domain (RBD) (MVA-RBD) (Fig. 1a). To evaluate the expression of the SARS-CoV-2 antigens from each vaccine vector, confluent monolayers of Vero cells were infected at a multiplicity of infection (MOI) of 10. At various timepoints after vector infection, infected cell supernatants and cell lysates were prepared for analysis by sodium dodecyl sulfate-polyacrylamide gel electrophoresis (SDS-PAGE) followed by Western blot using a rabbit polyclonal antibody to the SARS-CoV-2 spike protein.

Each vector expressed the expected species of S as early as 6 h after vector infection (Fig. 1b and Supplementary Fig. 1). Following MVA-S infection, the proteolytically processed S1 fragment was apparent in the supernatant and both full-length S and the processed S1 domain were present in the cell lysate (lanes 1 and 2). Infection with the MVA-ssM vector, which was engineered to express a soluble stabilized S ectodomain and had the S1/S2 cleavage site modified as previously described^6^, resulted in full-length S expression in both the supernatant and cell lysate (lanes 3 and 4). The much smaller SARS-CoV-2 RBD expressed by the MVA-RBD vector appeared in both the supernatant and the cell lysate of infected cells (lanes 5 and 6). It is not known why there is a slightly smaller RBD band apparent in the cell lysate, although it is possibly a result of additional proteolytic processing. Analysis of vector infected cells by confocal microscopy indicated that the majority of Spike protein expressed from MVA-S (full length) appeared to localize to both the cytoplasm and to the cell surface (Fig. 1c).

### Immunogenicity of MVA SARS-CoV-2 vectors in mice

**Fig. 2:**
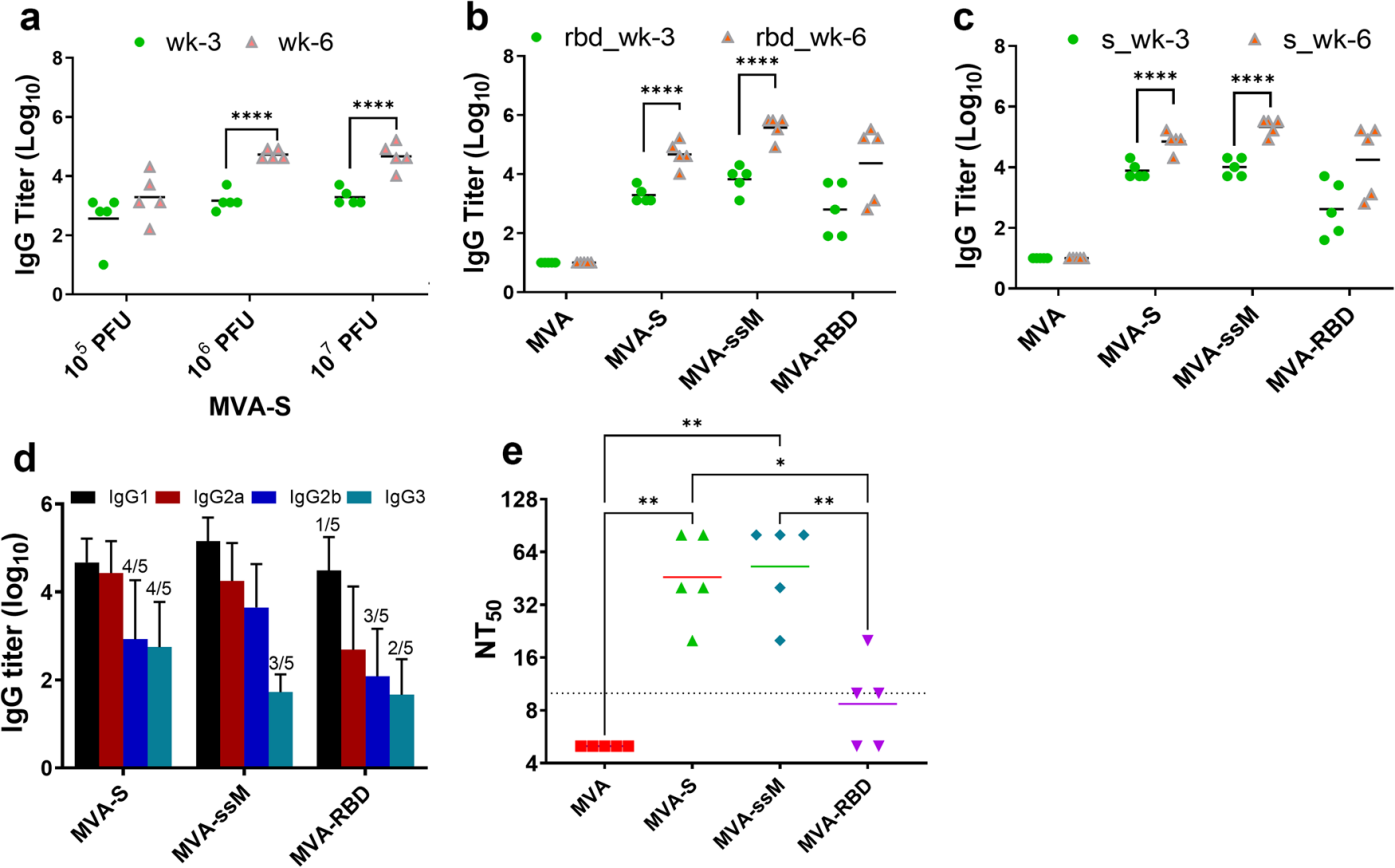
Antibody response to MVA SARS-CoV-2 vectors in mice. Mice were immunized subcutaneously with 10^5^, 10^6^, or 10^7^ PFU of MVA-S, MVA-ssM, MVA-RBD, or MVA (five5 per group) and boosted with the same vector dose at 3 weeks following the first immunization. **a** ELISA analysis of sera from MVA-S immunized mice using a full-length S capture antigen. **b** ELISA analysis of sera from mice immunized with all three MVA-S-expressing vectors at the 10^7^ PFU vector dose using RBD as a capture antigen. **c** ELISA analysis of sera from mice immunized with all three MVA-S-expressing vectors at the 10^7^ PFU vector dose using S as a capture antigen. **d** Sera from vector-immunized mice tested for antibody isotype by ELISA. Standard deviations are shown by error bars. Numbers above some bars (e.g., 4/5) indicate the number of seropositive mice of the total number of animals. **e** Neutralizing antibody analysis of sera from mice immunized twice with a 10^7^ PFU dose tested using a lentiviral vector pseudotype neutralization assay. *P < 0.1, **p < 0.01, and ****p < 0.0001.

Immunogenicity of the three MVA vaccine vectors MVA-S, MVA-ssM, and MVA-RBD was evaluated in dose-ranging studies in mice. Mice were immunized subcutaneously with 10^5^, 10^6^, and 10^7^ pore-forming unit (PFU) of each MVA vector or with empty MVA control (five per group) and boosted with the same vector dose at 3 weeks following the first immunization. Serum samples were obtained 3 weeks after each inoculation and tested for immunoglobulin M (IgM) and IgG response by enzyme-linked immunoassay (ELISA). S-specific IgM was not detected in sera samples at this timepoint, but ELISA analysis of sera from MVA-S-immunized mice using a full-length S capture antigen indicated that a SARS-CoV-2 spike-specific IgG antibody response was elicited by immunization that was dose-dependent and increased by a second immunization (Fig. 2a). A dose-dependent antibody response and a booster effect on antibody response were also observed in the ELISA analysis of sera from MVA-ssM and MVA-RBD immunized mice. At 10^5^ PFU, 10^6^ PFU, and 10^7^ PFU, the mean IgG titers ± SD after two immunizations with MVA-RBD were 3.11 ± 1.02, 3.59 ± 0.16, and 4.55 ± 1.07, respectively; similarly, the mean IgG titers ± SD after two immunizations with MVA-ssM were 5.27 ± 0.33, 5.40 ± 0.34, and 5.58 ± 0.39, respectively. Comparative ELISA results using both RBD and S as capture antigens with sera from mice immunized with all three MVA-S-expressing vectors at the 10^7^ vector dose indicated that all three vectors, but not the MVA control, elicited a strong antibody response and that the ELISA results using either capture antigen were similar (Fig. 2b, c).

Sera from vector-immunized mice were also tested for antibody isotypes (Fig. 2d). MVA-S elicited similar levels of IgG1 and IgG2a, as well as easily detectable IgG2b and IgG3 antibody isotypes. All antibody isotypes were also detected in MVA-ssM and MVA-RBD sera, although the IgG1/IgG2a ratio appeared higher in these sera. Taken together, the isotype results suggested a relatively balanced T-helper type 1 (Th1)/Th2 type response to vector immunization.

Finally, sera from mice immunized twice with a 10^7^ PFU dose were tested in a lentiviral vector pseudovirus-based neutralization assay (Fig. 2e). Neutralizing antibody was detectable in sera from three of the vector immunization groups, but not in the sera from the MVA-immunized control group. The strongest neutralizing antibody responses were measured in sera from the MVA-S- and MVA-ssM-immunized groups of mice; the response elicited to the MVA-RBD vector was noticeably lower and it is not clear what accounts for this difference, although it is possible that an MVA vector producing a secreted, non-anchored version of the RBD is not the optimal vector configuration.

### Evaluation of immunogenicity in Syrian hamsters

**Fig. 3:**
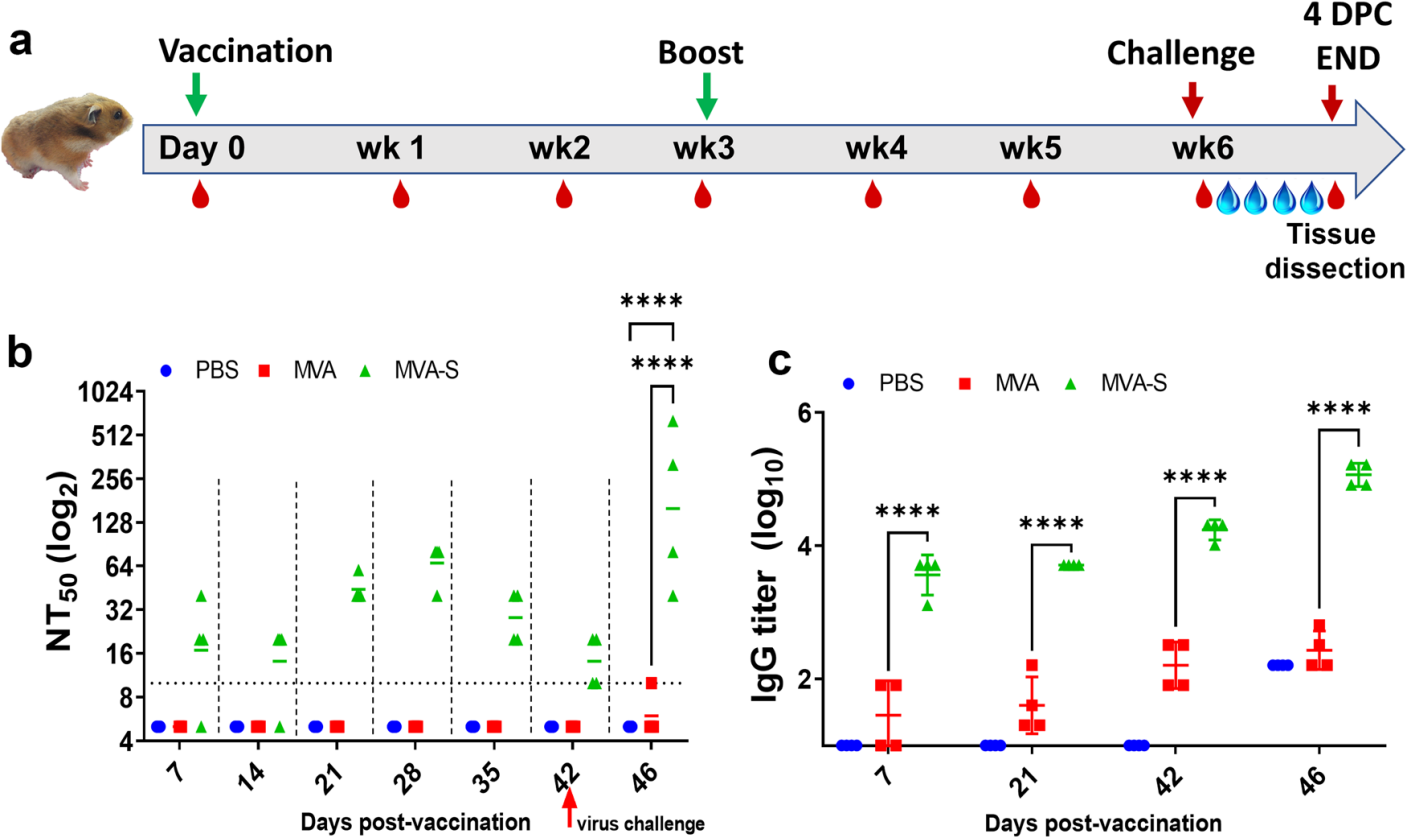
Experimental design and antibody responses to vaccination in Syrian hamsters. **a** Adult Syrian hamsters (*n* = 4) were vaccinated with MVA-S, MVA (empty vector), or PBS on days 0 and 21 with blood collection weekly until challenge in week 6. **b** Sera from collected blood samples were tested for neutralizing antibody at each week for 6 weeks and at 4 days post challenge. **c** Sera were tested for S-specific IgG at weeks 1, 3, and 6. ****P < 0.0001.

Based on the mouse immunogenicity results, we evaluated the immunogenicity and protective effect of the MVA-S vector in a Syrian hamster model as a baseline for comparison to other MVA vector constructions expressing different forms of S. Since the mouse results indicated a dose–response and a booster effect on antibody response, we employed both a single-dose and a prime-boost regimen with 10^8^ PFU (the largest practical dose available at the time of the experiments) on day 0 or days 0 and 21 (Fig. 3a). No adverse effects or clinical signs were observed as a result of MVA-S or MVA vaccination.

The ELISA antibody and neutralizing antibody response to S in hamsters were assessed in serum samples collected by weekly blood collection (gingival bleeding) post vaccination and once post challenge (Fig. 3a). Following a single immunization (prime-only), there were detectable neutralizing antibodies in some hamsters as early as 1 week post immunization and in all animals by 3 weeks post immunization (Fig. 3b, days 7 and 21). In the prime-boost immunization group, neutralizing antibodies were still detectable in week 6 just prior to challenge, but interestingly, the level of neutralizing antibody appeared somewhat lower than at 4 and 5 weeks post immunization (Fig. 3b, days 28, 35, and 42), although the level of antibody measured by ELISA remained very high at 6 weeks post immunization (Fig. 3c).

Importantly, at 5 days post challenge (DPC) a strong anamnestic response was observed in MVA-S-vaccinated animals in the prime-boost group where a ~50-fold increase in mean neutralizing titer was observed (Fig. 3b, day 47). In PBS and MVA-inoculated controls, seroconversion, as measured by neutralizing antibody, was not observed by 5 DPC.

### Evaluation of protection in Syrian hamsters

Three groups of Syrian hamsters that had received a single vaccination with MVA-S, MVA, or PBS were challenged with SARS-CoV-2 (WA1/2020, homologous with the S antigen expressed by MVA-S) at 3 weeks post immunization (Supplementary Fig. 2a). Neutralizing titers were low, and not detectable in all immunized animals, after a single immunization, but there was a >20-fold increase post challenge that was significantly greater than in the control groups (Supplementary Fig. 2b). Subgenomic RNA (replication) levels in nasal washes were similar in vaccinated hamsters and control hamsters at 1, 2, and 3 DPC, but lower in MVA-S-vaccinated hamsters at 4 DPC, although the differences were not significant (Supplementary Fig. 2c). Similarly, although not reaching statistical significance, viral loads in both nasal turbinates and lungs were lower in the MVA-S-vaccinated group than in the control groups (Supplementary Fig. 2d, e). In addition, reduced lung pathology in hamsters vaccinated with MVA-S compared to PBS or MVA-vaccinated hamsters was observed upon histopathology evaluation (Supplementary Fig. 2f). Thus, although the results suggested only a modest protective effect, the combined data from several types of the analysis indicated a measurable effect with a single immunization including a trend towards reduced lung pathology, reduced viral loads in nasal turbinates, nasal washes, and lungs at 4 DPC and a >20-fold increase in post-challenge NT50 titers.

**Fig. 4:**
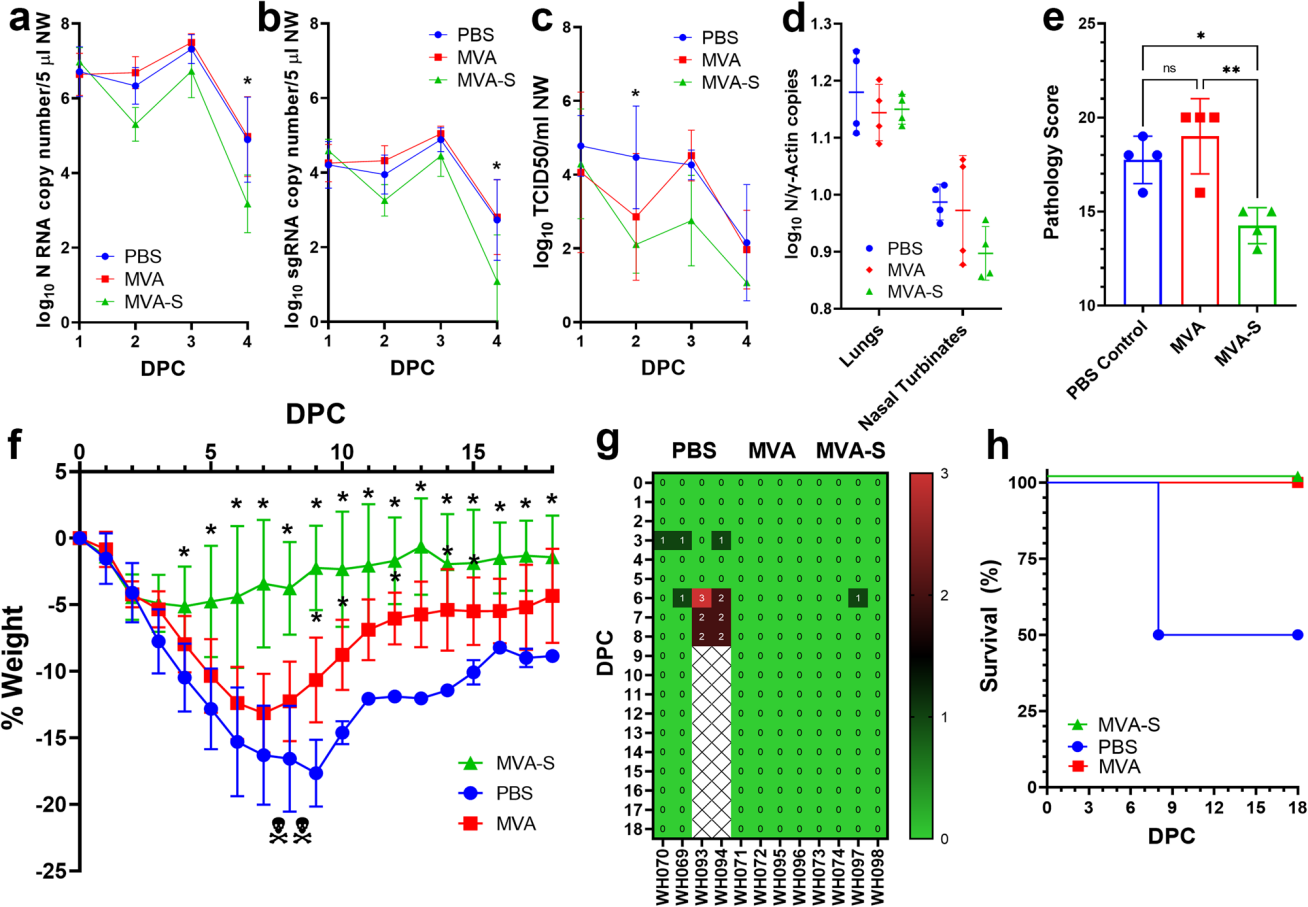
Efficacy of MVA-S vaccination in Syrian hamsters. Vaccinated and boosted adult Syrian hamsters (*n* = 4 per group) were challenged with 10^5^ TCID50 SARS-CoV-2 WA1/2020 in two experiments. In the first experiment, nasal washes were collected on days 1–4 post challenge (DPC) and titrated for total RNA (**a**), subgenomic RNA (**b**), and infectious virus titers by TCID50 assay (**c**). At 4 DPC, total viral RNA from lungs and nasal turbinates was quantified compared to γ-actin controls (**d**) and pathology in infected lungs compared by scoring (**e**). In a separate experiment, hamsters were vaccinated, boosted, and weighed daily for 18 days post challenge (**f**) with daily checks for morbidity (**g**) and mortality (**h**). Clinical scores (**g**) were assigned based on the following metric: 0—Normal; 1—Ruffled Fur; 1—Labored Breathing; 1—Lethargy; 1—Conjunctivitis. On day 8, two animals from the PBS control group met humane early endpoints, were euthanized, and removed from the study (). *P < 0.1, **p < 0.01, and n.s. not significant. Standard deviations are shown by error bars.

**Fig. 5:**
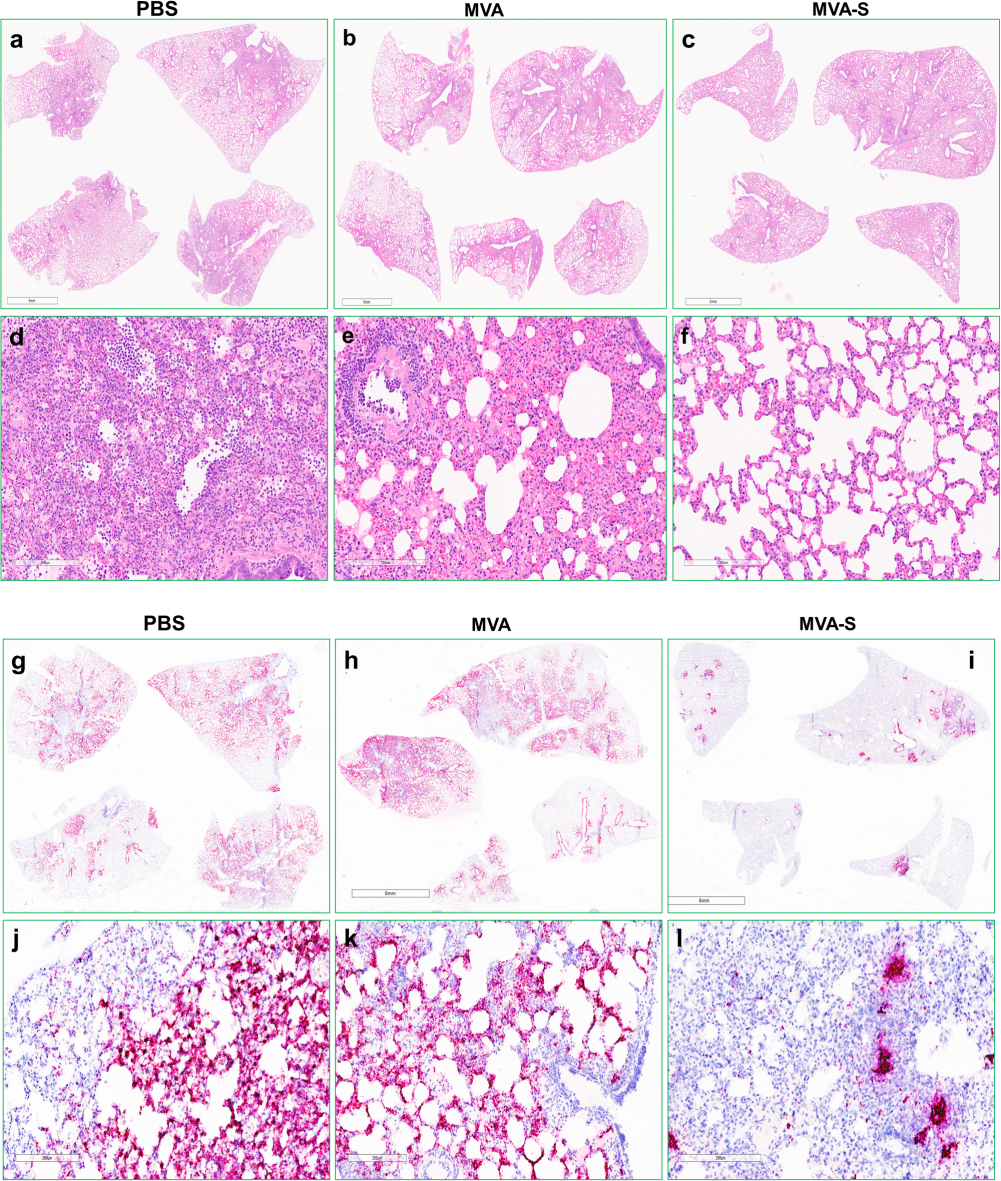
Histopathology and detection of SARS-CoV-2 RNA in the lungs of challenged Syrian hamsters. Lung tissues were harvested at 4 days post SARS-CoV-2 challenge from hamsters that were previously vaccinated with PBS, MVA, or MVA-S. Formalin-fixed, paraffin-embedded lung tissues were stained either by hematoxylin and eosin (H&E, **a**–**f**) or by a SARS-CoV-2 RNAscope probe (**g**–**l**). Pathology scores were determined by a licensed pathologist who viewed 3–4 sections in each study group in a blinded fashion. Panels **a**–**c** and **g**–**i** are images of all lobes from a representative hamster in each group. Panels **d**–**f** and **j**–**l** are close-up images from a representative area of panels **a**–**c** and **g**–**i**, respectively. In panels **g**–**l**, the blue color indicates nucleus staining and the red/pink color represents SARS-CoV-2 spike positive-sense (genomic) RNA. The scale bar represents 5 mm (**a**–**c**); 6 mm (**g**–**i**); and for the rest (**d**–**f**, **j**–**l**) the bar represents 200 μm.

We next conducted a pair of prime-boost vaccination experiments in an effort to improve immunogenicity and protection. In the first experiment, hamsters were followed for 4 days before sacrifice and histopathology evaluation. Nasal washes were collected daily on 1–4 DPC and titrated for viral RNA (vRNA) and infectious titers to detect viral shedding in the upper respiratory tract. Overall, viral load and pathology were reduced in MVA-S-vaccinated hamsters after a prime and boosting dose (Fig. 4). Both total vRNA and subgenomic RNA (replication) levels in nasal washes were lower in vaccinated hamsters than control hamsters at 2, 3, and 4 DPC. At 4 DPC total vRNA (*p* = 0.0016, Dunnett’s multiple comparison test) and subgenomic RNA (*p* = 0.0030, Dunnett’s multiple comparison test) levels were significantly reduced in the nasal washes of MVA-S vaccinated hamsters compared to PBS controls by ~40-fold (Fig. 4a, b). Infectious virus titers in nasal washes, as measured by TCID_50_, were lower in MVA-S-vaccinated hamsters at all timepoints, and were significantly reduced at 2 DPC in MVA-S-vaccinated hamsters by >100-fold (*p* = 0.0218) compared to controls. All animals (*n* = 4) were euthanized at 4 DPC and the lungs (right cranial lobe) and nasal turbinates were collected for titration of viral loads by real-time quantitative reverse transcription-PCR (qRT-PCR) (Fig. 4d). Similar levels of total vRNA, compared to γ-actin RNA levels, were measured in lungs; lower levels of total vRNA were observed in nasal turbinates from MVA-S-vaccinated hamsters, although the differences did not reach statistical significance. The remainder of the lungs were scored for pathology (Fig. 4e) with MVA-S-vaccinated animals showing protection from viral challenge. The reduction in overall clinical score was largely contributed by less severe or nonexistent manifestations of atypical pneumocyte hyperplasia, hemorrhage, vasculitis, and especially consolidation (Fig. 4e). The most commonly observed pathologies in mock- and MVA-vaccinated hamsters challenged with SARS-CoV-2 infection were alveolar wall thickening, alveolar airway infiltrates, perivascular infiltrates, perivascular edema, type II pneumocyte hyperplasia, atypical pneumocyte hyperplasia, bronchiole mucosal hyperplasia, bronchiole airway infiltrates, proteinaceous fluid, and consolidation (Fig. 5). Overall, total pathology scores were significantly reduced in MVA-S-vaccinated hamsters compared to mock-vaccinated (*p* = 0.0206, Tukey’s multiple comparisons test) or MVA-vaccinated (*p* = 0.0035, Tukey’s multiple comparisons test) groups (Fig. 4e). Severe alveolar airway infiltration by neutrophils, foamy macrophages, and lymphocytes was observed in all three groups including MVA-S-vaccinated hamsters as was mild to moderate perivascular edema and moderate perivascular infiltration composed of lymphocytes, plasma cells, and macrophages. In addition to measuring viral loads in lungs by qRT-PCR, probes specific to SARS-CoV-2 spike genomic RNA were used to visualize virus particles by in situ hybridization (ISH) using RNAscope™ technology (Fig. 5). The ISH agreed with quantification of viral loads, with less observable virus staining in the lungs of MVA-S-vaccinated hamsters compared to the PBS or MVA-vaccinated controls.

In the second challenge experiment where hamsters received both a prime and boosting dose of PBS, MVA, or MVA-S, we followed weight loss (Fig. 4f), clinical signs (Fig. 4g), and mortality (Fig. 4h) for 18 DPC. Significant differences in weight loss were observed on days 4–14 and 16–18 between mock-vaccinated (PBS) and MVA-S-vaccinated animals using Dunnett’s multiple comparison test. Vaccination with MVA alone was also associated with some reduction in weight loss in challenged hamsters, which was less pronounced than in MVA-S-vaccinated hamsters, but was nonetheless significant. In the PBS-vaccinated control group, two animals were euthanized due to morbidity (extreme lethargy, labored breathing) at 8 DPC (Fig. 4h). Taken together, the data indicate that immunization of hamsters with two doses of an MVA vector expressing SARS-CoV-2 S antigen provided significant protection against a SARS-CoV-2 virus challenge.

## Discussion

In the study reported here, we constructed MVA vectors expressing full-length SARS-CoV-2 spike protein (MVA-S), as well as a soluble form of the S ectodomain and the RBD of the N-terminal subunit S1, and evaluated these vectors for immunogenicity and protective effect in mouse and hamster models. The goal of these studies was to begin a systematic comparison and evaluation of the various forms of virus vector expressed S antigen using the well-characterized MVA vector platform. Although MVA vectors are known to elicit both humoral and cellular immunity to expressed antigens, we focused on the antibody response to S in this study due to accumulated evidence of the important role that antibody to S plays in protection. Of course, the relative role of antibodies in the protective response elicited by vaccination will undoubtedly depend on the nature of the particular vaccine.

All three MVA vectors expressing the forms of S evaluated in this study elicited a SARS-CoV-2-specific antibody response when used to immunize mice, although characterization of the antibody response revealed some interesting differences. Interestingly, although the ELISA antibody response to S, soluble S, and the RBD antigen was similar in mice, the neutralizing antibody response to the RBD expressing vector was noticeably lower than that elicited by the MVA-S or MVA-ssM vectors. Since the majority of neutralizing antibodies to S has been shown to be directed to epitopes in the RBD^7^, this observation probably reflects differences in the presentation of the RBD antigen in the three vectors.

Several forms of the spike glycoprotein have been incorporated into various SARS-CoV-2 candidate vaccines, including versions of S designed to stabilize the prefusion conformation in order to increase the stability of recombinant S as well as to enhance immunogenicity. Spike variants modified to contain prolines at positions 986 and 987 (S-2P) were used to generate soluble stable ectodomain forms of S for structural analysis^1,6^ and have been incorporated into many vaccine candidates, along with modification of the S1/S2 cleavage site. More recently, additional stabilizing mutations have been described that may further enhance the production of spike protein^8^. For the construction of an MVA vector expressing a soluble ectodomain form of S, we incorporated the key features of S-2P with the expectation that these features would be critical for a stable soluble S. For the construction of an MVA vector expressing full-length S, however, we chose to insert a full-length unmodified form of S from an early isolate of SARS-CoV-2 so that this vector can be used as a baseline to compare to additional vector constructions both for immunogenicity as well as protection.

The emergence of SARS-CoV-2 into the human population and subsequent development of the ongoing pandemic resulted in an immense expedited effort to develop relevant animal models to study virus pathogenesis and to assess vaccine candidates. Currently, there are several viable animal models, including non-human primates, mice, and hamsters, that reproduce at least some of the defining characteristics of SARS-CoV-2 pathogenesis, and as such, can be utilized to evaluate immunogenicity and protection afforded by candidate vaccines (for a recent review, see ref. ^9^). We have previously reported that aged hamsters can develop more pronounced disease and even fatality upon challenge, thus recapitulating key aspects of SARS-CoV-2 infection in humans^10^. Although Syrian hamsters (*Mesocricetus auratus*) can live to be 18–24 months old and reach sexual maturity after ~12–13 weeks of age, studies frequently employ 5–10-week-old hamsters^11,12^ that are more logistically viable but immature. As advanced age strongly correlates with severe disease in COVID-19 patients, aged hamsters support higher levels of viral replication than younger hamsters and develop severe pneumonia after infection^10^. We chose to use adult Syrian hamsters (>10 months old) to model vaccine protection in an adult human population, as children (although susceptible to infection) do not tend to show severe symptoms^13^.

The results from the current study demonstrate that an MVA vector expressing a full-length version of S provided protection in aged hamsters challenged with SARS-CoV-2 as evidenced by several measures including viral loads, weight loss, and lung histopathology. One immunization with the MVA-S vector elicited low levels of antibody to S, but this immunization clearly primed the animals since the measured neutralizing antibody titers in the MVA-S group were significantly higher than in the two control groups at 4 DPC. Notably, even after one immunization, which presumably was suboptimal based on viral load measurements and histopathology, there was no predisposition towards enhanced disease upon challenge. Two immunizations with the MVA-S vector elicited higher levels of antibody to S as measured by ELISA and neutralization, and there was increased evidence of protection, particularly in measurements of animal weight loss and in lung histopathology scores. Interestingly, immunization with an MVA vector alone was associated with some reduction in weight loss following the challenge and there were no deaths in the challenged MVA vector group. Similar observations of heterologous protection afforded by certain live vaccines have been noted, and, in fact, the use of such vaccines as a potential tool to mitigate the severity of SARS-CoV-2 infection has been proposed^14^.

In addition to the adenovirus vector vaccines that have entered large-scale phase 3 clinical trials (reference), several other types of viral vectors have been developed and are being evaluated as potential candidate SARS-CoV-2 vaccines including MVA vector vaccines^15–18^. MVA is a currently licensed vaccine for smallpox and monkeypox (https://www.fda.gov/vaccines-blood-biologics/jynneos) and candidate MVA vector vaccines have been developed and evaluated for numerous pathogens including other coronaviruses such as SARS-CoV and MERS^2,3^. Consequently, there exists a fairly extensive safety database for MVA in humans, as well as ample evidence for the immunogenicity of MVA vectors. The immunogenicity of MVA vectors expressing forms of SARS-CoV-2 S in mice has been described recently^15–18^, and in general, the data from all reported studies indicate that such MVA vectors elicit both S-specific antibody and S-specific T cell responses, although as noted earlier, the relative contribution of humoral and cellular immune responses to protection for a particular vaccine or animal model is unknown. However, in addition to the full-length unmodified S antigen used in some other studies, we also evaluated in mice MVA vectors expressing a soluble secreted form of the S ectodomain and a non-anchored version of the RBD region of S, demonstrating that the soluble form of S, but not the RBD, elicited levels of neutralizing antibody similar to that of full-length S. The results from our mouse studies suggest that additional hamster challenge studies comparing the protective effects of such MVA vectors is warranted. Finally, the study by Liu et al. also included immunogenicity assessments in mice of MVA vectors expressing stabilized forms of full-length S as well as other forms of S such as an anchored RBD^17^. Taken together, the data suggest that as SARS-CoV-2 vaccine development continues, it will be important to continue to evaluate and optimize the forms of S to be expressed as vaccine antigens.

Two studies with MVA SARS-CoV-2 vectors have reported the effectiveness of such vectors in protecting transgenic mice expressing human ACE2 (hACE2)^15,17^ and another study reported effectiveness in a mouse model sensitized with a human ACE2-expressing adenovirus^18^. Our study extends these findings from mouse models of protection by incorporating an adult hamster model of challenge. In contrast to the mouse challenge model, a single MVA-S vector immunization of hamsters provided only a modest protective effect, but two immunizations provided significant protection against a strong SARS-CoV-2 challenge. These apparent differences may be due to slightly different forms of vectors and expressed S antigen, but may also be due to differences in the animal challenge models. There are advantages and limitations to all animal challenge models. For example, aged hamsters replicate multiple disease manifestations in common with human patients including lung pathology and virus shedding, but there are differences in the virus pathology in the two animal models and there are practical limitations to the number of animals that can be employed in hamster experiments compared to mouse experiments. Regardless, the use of multiple animal models is complementary and the results of all studies to date provide strong support for the continued development of MVA SARS-CoV-2 vaccine vectors.

In summary, the results of several hamster challenge studies taken together indicate that immunization with two doses of a prototype MVA vector expressing S antigen provides significant protection against a SARS-CoV-2 virus challenge and reaffirm the utility of this animal model for evaluating candidate SARS-CoV-2 vaccines. The results from the present study should facilitate the development of vaccine vectors that express optimized forms of SARS-CoV-2 S antigens and inform vaccine development for almost any vaccine vector platform.

## Methods

### Cells and viruses

UMNSAH/DF-1 cells (ATCC CRL 12203) and Vero cells (ATCC CRL 1586) were obtained from the American Type Culture Collection and were maintained and passaged in Dulbecco’s modified Eagle’s medium (Gibco/Invitrogen Life Technologies, Carlsbad, CA) supplemented with 10% fetal bovine serum (Hyclone). MVA was originally obtained from Dr. Bernard Moss (NIAID, National Institutes of Health, Bethesda, MD), which was amplified and titrated in DF-1 cells.

The SARS-CoV-2 isolate WA1/2020 (NR-52281, lot. 70033175) was obtained from BEI Resources, NIAID, NIH, and had been passed three times on Vero cells and one time on Vero E6 cells prior to acquisition. It was further passed once on Vero E6 cells in our laboratory. The virus was sequence-verified to contain no mutation to its original seed virus.

### Construction of MVA shuttle plasmids

Recombinant MVA vectors were designed and constructed to express full-length SARS-CoV-2 spike protein (MVA-S), a soluble spike ectodomain (MVA-ssM), or the SARS-CoV-2 RBD (MVA-RBD). Sequences were codon optimized for expression in mammalian cells, including a substitution of the sequence encoding the SARS-CoV-2 spike protein signal peptide with the sequence encoding the signal peptide of the human immunoglobulin heavy chain (GenBank # QBK47409.1). The sequences were corrected for cryptic poxvirus transcription termination signals and inserted into the *I8R*-*G1L* intergenic region of the MVA genome by homologous recombination. The full-length S protein (USA-WA1/2020; GenBank accession # MN985325.1) was synthesized and cloned into pUC57 (GenScript, Piscataway, NJ) to generate the plasmid pUC57-S. Similarly, pUC57-S_ssT contained a synthetic SARS-CoV-2 sequence in which the sequence encoding the transmembrane domain and cytoplasmic tail was replaced with the sequence encoding a trimerization (foldon) sequence along with nucleotide substitutions that altered the S1/S2 protease cleavage site from residues RRAR to GSAS and residues 986/987 from KV to PP^6^. The two plasmids were used as templates for the amplification of sequences encoding spike protein forms (Table 1) for cloning into the MVA shuttle vector/Destination vector, pCAM18^19^. PCR amplicons were initially cloned into pENTR-D-TOPO vector (Invitrogen, Carlsbad, CA) to obtain pENTR-S, pENTR-ssM, and pENTR-RBD. The pENTR clones were sequenced to confirm the authenticity of the inserted SARS-CoV-2 spike sequences and authenticated pENTR clones were recombined with pCAM18 using the Gateway LR Clonase Enzyme mix (Invitrogen, Carlsbad, CA) to generate Shuttle plasmids pCAM18-S, pCAM18-ssM, and pCAM18-RBD.

To generate the MVA shuttle vector, primers CM-175 and CM-174 were used to amplify S from plasmid pUC57-S, primers CM-175 and CM-186 were used to amplify ssM from plasmid pUC57-ssT, and primers CM-187 and CM-188 were used to amplify RBD from plasmid pUC57-S.

The PCR primer sequences are as follows:

CM-174: 5ʹ-CAGGTCGACTCTAGTCAGGTGTAGTGCA

CM-175: 5ʹ-CACCGGATCCTCACCATGGGCTGGAGCTGCAT

CM-186: 5ʹ-CTCAGTGGTGGTGGTGGTGGTGCTGCTCGTACTTGCCCAGCTCCTGCAGGTCGAT

CM-187: 5ʹ-CACCATGGGCTGGAGCTGCATCATCCTGTTCCTGGTGGCCACCGCCACCGGCGTGCACAGCCAGTGCAGGGTGCAGCCCACCGAGAGCATCGTGAGGTT

CM-188: 5ʹ-GTCAGTGGTGGTGGTGGTGGTGGCCCAGGTTGGTGCTCTTCTTGGGGCCGCACA

### Construction of recombinant MVA vectors

MVA vectors were constructed as previously described^19^. Briefly, confluent monolayers of DF-1 cells in a 6-well tissue culture plate were infected with MVA at a multiplicity of infection (MOI) of 0.1 and transfected with 1 µg of a shuttle plasmid, using X-tremeGENE HP DNA transfection reagent (Sigma-Aldrich, St. Louis, MO). Infected/transfected cells were harvested after 48 h. About 70% of each cell harvest volume was centrifuged, and cell pellets were lysed in RIPA buffer for Western blot analysis to identify clones expressing the spike form of interest. The remaining cell harvest (~30%) was used for the subsequent isolation of recombinant MVA.

Recombinant MVA vectors were isolated by three to five serial plaque purifications on DF-1 cells; purified recombinant MVA vectors were expanded in DF-1 cells. Recombinant vectors for animal inoculation were further purified on 36% sucrose cushion as previously described^20,21^ and titrated in DF-1 cells.

### Evaluation of SARS-CoV-2 antigen expression by Western blot analysis and immunofluorescence

Confluent monolayers of Vero cells in 6-well tissue culture plates were infected at an MOI of 10 with purified MVA-S, MVA-ssM, or MVA-RBD, or with empty MVA. Supernatants and infected cells were harvested at 6 h post infection. The supernatant in each well was clarified and concentrated to between 50 and 70 µL in 0.5 mL concentrators (10 kDa molecular weight cut-off; Pierce). The infected cells were scrapped, pelleted by centrifugation, and each cell pellet was lysed in 100 µL RIPA buffer containing a cocktail of protease inhibitors (SIGMAFAST Protease Inhibitor Cocktail; Sigma-Aldrich, St. Louis, MO), followed by three freeze/thaw cycles. Western blot analysis was performed as previously described^21^. Briefly, proteins in supernatant concentrate and cell lysates were resolved by reduced SDS-PAGE in a 12-well 4–12% NUPAGE Bolt gel and iBlotted onto a nitrocellulose membrane. The membrane was incubated with rabbit anti-SARS-CoV-2 spike protein polyclonal antibody (MyBioSource, San Diego, CA) at 1:1000 for 1.5 h, followed by incubation with a goat anti-rabbit antibody (horseradish peroxidase (HRP) conjugate; SouthernBiotech, Birmingham, AL) at 1:5000 dilution. The blot was developed by adding the Supersignal West Dura HRP substrate (Pierce). Images were captured under the LAS3000 Imaging System (Fujifilm, Tokyo, Japan).

For immunofluorescence assay, Vero E6 cells were plated on collagen-coated glass coverslips the day before infection into 24-well plates at 5 × 10^4^ cells/well. Cells were infected with MVA or MVA-S at an MOI of 1 for 90 min before the medium change. Infected cells were incubated for an additional 16 h and then fixed in 4% paraformaldehyde for 15 min at room temperature. To permeabilize, 0.2% Triton X-100 in PBS was added to each well for 15 min, followed by three washes with 1× phosphate-buffered saline (PBS). Cells were then stained in anti-SARS-CoV-2 antibody at 1:200 dilution overnight, followed by a secondary antibody staining (1:5000 Alexa goat anti-rabbit IgG 568). Images were captured with a Leica Stellaris confocal microscope.

### Animals, immunizations, and challenges

Six- to eight-week-old male BALB/cByJ mice were purchased from the Jackson Laboratories (Bar Harbor, ME) and housed in cages at a core facility at CBER/FDA. Sterile food and water were supplied ad libitum. All immunizations and blood draws were performed in accordance with an animal protocol approved by the FDA White Oak Consolidated Animal Program (#2008-02); procedures were similar to those described previously^22^. Briefly, mice in treatment groups (five per group) were inoculated with 10^5^, 10^6^, and 10^7^ PFU of each MVA vector diluted in Dulbecco’s PBS. For each experiment, a control group was inoculated with 10^7^ PFU of empty MVA vector or with PBS. All inoculations were via the subcutaneous route as previously described^22^. Mice were inoculated twice at an interval of 3 weeks. Serum samples obtained 3 weeks after each inoculation were tested for total IgG antibody responses in ELISA, using recombinant baculovirus-expressed full-length S and RBD antigens.

All hamsters were housed and challenged by the intranasal route as described previously^23^ (FDA White Oak Consolidated Animal Program [#2020-06]). Adult (aged 10–20 months) male and female Syrian hamsters (*Mesocricetus auratus*) were vaccinated by the subcutaneous route with 10^8^ PFU of MVA-S, empty vector (MVA), or PBS as a negative control (*n* = 4 per group). Blood was collected weekly post vaccination and centrifuged to collect serum samples. In experiments employing a booster vaccination, animals were vaccinated a second time at 3 weeks after the initial vaccination with the same route and dose of inoculum.

### Histopathology analyses

Tissues were fixed in 10% neural-buffered formalin overnight and then processed for paraffin embedding. The 4-μm sections were stained with hematoxylin and eosin for histopathological examinations. Images were scanned using an Aperio ImageScope on an AT2 scanner. The pathology was graded blinded for the following 12 categories: consolidation, alveolar wall thickening, alveolar airway infiltrates, perivascular infiltrates, perivascular edema, type II pneumocyte hyperplasia, atypical pneumocyte hyperplasia, bronchiole mucosal hyperplasia, bronchiole airway infiltrates, proteinaceous fluid, hemorrhage, vasculitis. Grading: 0 = none, 1 = mild, 2 = moderate, and 3 = severe. Each pathology category was weighted equally as previously described^24^. A graph was prepared by summing up the score in each category.

### Measurement of antibody by ELISA

SARS-CoV-2 S and RBD antigens for ELISA were prepared in a baculovirus expression system. The synthetic DNA sequence encoding the S ectodomain (16-1208) was cloned downstream of the sequence encoding the GP67 signal peptide and upstream of the sequences for the T4 phage foldon trimerization signal and 6xHis-tag; the DNA sequence encoding the RBD (319–533) was cloned downstream of the GP67 signal peptide and upstream of a 6xHis-tag. Recombinant bacmids were generated and then used to produce infectious recombinant baculovirus stock that was then used to infect insect cells for protein expression. The supernatant was harvested 72–96 h post infection and recombinant His-tag-containing protein purified using Ni-NTA resin. Gel filtration (Superdex-200) was used for additional purification. Recombinant proteins were stored at −80 °C in 100 mM Tris (pH 8)/150 mM NaCl/10% glycerol.

Quantitation of antibody responses was performed by enzyme-linked immunosorbent assay as previously described^21^. Briefly, Immunlon 2HB plates were coated with recombinant S or RBD protein at 1 µg/mL overnight at 4 °C. Test serum samples were pre-diluted in assay diluent (PBS containing 0.05% Tween-20 [PBST] and 10% fetal bovine serum), followed by serial two-fold dilutions of each sample in duplicates across the plate. Plates were incubated with the test serum samples for 2 h at 37 °C. After rigorous plate washes in a microplate washer, a secondary antibody (anti-mouse IgG or anti-hamster IgG) conjugated to HRP (SouthernBiotech, Birmingham, Alabama) was added to wells at 1:5000 dilution (mouse IgG ELISA) or 1:4000 dilution (hamster IgG ELISA). For IgG isotype assays, each isotype (anti-mouse IgG1, IgG2a, IgG2b, and IgG3) was used at a 1:3000 dilution. Plates were incubated with secondary antibody for 1 h, washed, and ABTS/H_2_O_2_ peroxidase substrate (SeraCare, Gaithersburg, MD) was added to assay wells. After 20 to 30 min at ambient temperature, reactions were stopped with 1% SDS, and OD_405_ values were captured on the Versamax microplate reader with the Softmax Pro 7 software installed (Molecular Devices, San Jose, CA). The assay endpoint was a mean OD_405_ of 0.05 for duplicate wells for the full-length S ELISA and a mean OD_405_ of 0.01 for the RBD ELISA. The reciprocal of the highest serum dilution at which the mean OD_405_ value averaged ≥0.05 (full-length S ELISA) or ≥0.01 (RBD ELISA) was the IgG titer.

Testing of hamster serum samples for total IgG antibody responses was similar to the one described above. However, background subtraction was applied to the hamster IgG ELISA by subtracting the mean OD_405_ values for the PBS treatment group from the mean OD_405_ readings for other treatment groups. The assay endpoint was a mean OD_405_ of 0.05 for duplicate wells for both full-length S and RBD ELISAs, after background subtractions. The reciprocal of the highest serum dilution at which the mean OD_405_ value averaged ≥0.05 after background subtractions was the hamster anti-S or anti-RBD IgG titer.

### Antibody neutralization

Neutralization assays using pseudotyped virions were performed as described previously^10^. Briefly, human codon-optimized complementary DNA (cDNA) encoding SARS-CoV-2 S glycoprotein (NC_045512) was synthesized by GenScript and cloned into eukaryotic cell expression vector pcDNA 3.1 between the *Bam*HI and *Xho*I sites. Pseudovirions were produced by co-transfection of Lenti‐X 293T cells with psPAX2, pTRIP-luc, and SARS-CoV-2 S-expressing plasmid using Lipofectamine 3000. The supernatants were harvested at 48 and 72 h post transfection and filtered through 0.45-mm membranes prior to use. For neutralization assays, 50 µL of SARS-CoV-2 S pseudovirions were preincubated with an equal volume of medium containing serum at varying dilutions at room temperature for 1 h, and then virus–antibody mixtures were added to Vero E6 cells in a 96-well plate. After a 3 h incubation, the inoculum was replaced with a fresh medium. Cells were lysed 48 h later, and luciferase activity was measured using a luciferin-containing substrate. Controls included cells only, viruses without any antibody, and positive-control sera. The end-point titers were calculated as the last serum dilution resulting in at least 50% SARS-CoV-2 neutralization. The amount of pseudovirions used in the assay was determined to give rise to a target input of 5 × 10^5^–10^7^ RLU/ml.

### Immunohistochemistry and ISH

Lung samples were fixed in 10% neural-buffered formalin overnight and then processed for paraffin embedding as previously described^23^. The 4 μm sections were stained with hematoxylin and eosin for histopathological examinations. Images were scanned using an Aperio ImageScope. To detect SARS-CoV-2 genomic RNA in FFPE tissues, ISH was performed using the RNAscope 2.5 HD RED Kit, a Singleplex assay method (Advanced Cell Diagnostics; catalog 322373) following the manual according to the manufacturer’s instructions. Briefly, Mm PPIB probe detecting peptidylprolyl isomerase B gene (housekeeping gene) (catalog 313911, positive-control RNA probe), dapB probe detecting dihydrodipicolinate reductase gene from *Bacillus subtilis* strain SMY (a soil bacterium) (catalog 310043, negative-control RNA probe), and V-SARS-Cov-2-S (catalog 854841) targeting SARS-CoV-2 spike positive-sense (genomic) RNA were used to carry out the assay. Tissue sections were deparaffinized with xylene, underwent a series of ethanol washes and peroxidase blocking, and were then heated in kit-provided antigen retrieval buffer and digested by kit-provided proteinase. Sections were exposed to ISH target probes and incubated at 40 °C in a hybridization oven for 2 h. After rinsing, the ISH signal was amplified using a kit-provided preamplifier and amplifier conjugated to alkaline phosphatase and incubated with a fast-red substrate solution for 10 min at room temperature. Sections were then stained with 50% hematoxylin solution, followed by 0.02% ammonium water treatment, dried in a 60 °C dry oven, mounted, and stored at 4 °C until image analysis.

### SARS-CoV-2 genomic and subgenomic RNA quantification

RNA was extracted from 50 µL nasal wash or 0.1 g tissue homogenates using QIAamp Viral RNA Mini Kit or the RNeasy 96 Kit (Qiagen) and eluted with 60 μL of water. Five microliters of RNA was used for each reaction in real-time RT-PCR. Quantification of SARS-CoV-2 vRNA was conducted using the SARS-CoV-2 (2019-nCoV) CDC qPCR Probe Assay (IDTDNA) using iTaq Universal Probes One-Step Kit (Bio-Rad). The standard curve was generated using 2019-nCoV_N_Positive Control (IDTDNA). The detection limit of the vRNA was determined to be 100 copies/reaction. Quantification of SARS-CoV-2 E gene subgenomic mRNA (sgmRNA) was conducted using Luna Universal Probe One-Step RT-qPCR Kit (New England Biolabs) on a Step One Plus Real-Time PCR System (Applied Biosystems). The primer and probe sequences were: SARS2EF, CGATCTCTTGTAGATCTGTTCT; PROBE, FAM-ACACTAGCCATCCTTACTGCGCTTCG-BHQ-1; SARS2ER, ATATTGCAGCAGTACGCACACA. To generate a standard curve, the cDNA of SARS-CoV-2 E gene sgmRNA was cloned into a pCR2.1-TOPO plasmid. The copy number of sgmRNA was calculated by comparing to a standard curve obtained with serial dilutions of the standard plasmid. The detection limit of the sgmRNA was determined to be 25 copies/reaction. Values below detection limits were arbitrarily set to half of the lower detection limit for graphing purposes.

### Statistical analysis

Differences in antibody responses between treatment groups were compared using a statistical *t* test of the log 10-transformed IgG titers, one-way analysis of variance, or the Mann–Whitney rank-sum test where an initial test of normality failed. In all cases, a *p* value < 0.05 was considered statistically significant. Statistical tests were performed using GraphPad Prism version 9.0.0 for Windows (GraphPad Software, San Diego, CA, USA; www.graphpad.com).

### Reporting summary

Further information on research design is available in the Nature Research Reporting Summary linked to this article.

## Supplementary information

Reporting Summary

Supplementary Information

## Data Availability

The authors declare that all relevant data supporting the findings of this study are available within the paper and its Supplementary information files.
