# Supplementary material for: MVA vector expression of SARS-CoV-2 spike protein and protection of adult Syrian hamsters against SARS-CoV-2 challenge
Source: NPJ Vaccines. 2021 Dec 3;6:145. doi: 10.1038/s41541-021-00410-8 (PMC8642471; doi:10.1038/s41541-021-00410-8)
Supplement: Supplementary file 1 — Reporting Summary [file 41541_2021_410_MOESM1_ESM.pdf]

nature portfolio

Corresponding author(s): Jerry P Weir

Last updated by author(s): Sep 22, 2021

Reporting Summary

Nature Portfolio wishes to improve the reproducibility of the work that we publish. This form provides structure for consistency and transparency in reporting. For further information on Nature Portfolio policies, see our Editorial Policies and the Editorial Policy Checklist.

Statistics

For all statistical analyses, confirm that the following items are present in the figure legend, table legend, main text, or Methods section.

n/a	Confirmed	
☐	☒	The exact sample size (*n*) for each experimental group/condition, given as a discrete number and unit of measurement
☐	☒	A statement on whether measurements were taken from distinct samples or whether the same sample was measured repeatedly
☐	☒	The statistical test(s) used AND whether they are one- or two-sided<br>*Only common tests should be described solely by name; describe more complex techniques in the Methods section.*
☒	☐	A description of all covariates tested
☒	☐	A description of any assumptions or corrections, such as tests of normality and adjustment for multiple comparisons
☐	☒	A full description of the statistical parameters including central tendency (e.g. means) or other basic estimates (e.g. regression coefficient) AND variation (e.g. standard deviation) or associated estimates of uncertainty (e.g. confidence intervals)
☐	☒	For null hypothesis testing, the test statistic (e.g. *F*, *t*, *r*) with confidence intervals, effect sizes, degrees of freedom and *P* value noted<br>*Give P values as exact values whenever suitable.*
☒	☐	For Bayesian analysis, information on the choice of priors and Markov chain Monte Carlo settings
☒	☐	For hierarchical and complex designs, identification of the appropriate level for tests and full reporting of outcomes
☒	☐	Estimates of effect sizes (e.g. Cohen's *d*, Pearson's *r*), indicating how they were calculated

Our web collection on statistics for biologists contains articles on many of the points above.

Software and code

Policy information about availability of computer code

Data collection	*Provide a description of all commercial, open source and custom code used to collect the data in this study, specifying the version used OR state that no software was used.*
Data analysis	*Provide a description of all commercial, open source and custom code used to analyse the data in this study, specifying the version used OR state that no software was used.*

For manuscripts utilizing custom algorithms or software that are central to the research but not yet described in published literature, software must be made available to editors and reviewers. We strongly encourage code deposition in a community repository (e.g. GitHub). See the Nature Portfolio guidelines for submitting code & software for further information.

Data

Policy information about availability of data

All manuscripts must include a data availability statement. This statement should provide the following information, where applicable:

- Accession codes, unique identifiers, or web links for publicly available datasets
- A description of any restrictions on data availability
- For clinical datasets or third party data, please ensure that the statement adheres to our policy

The authors declare that all relevant data supporting the findings of this study are available within the paper and its supplementary information files.

Field-specific reporting

Please select the one below that is the best fit for your research. If you are not sure, read the appropriate sections before making your selection.

☒ Life sciences ☐ Behavioural & social sciences ☐ Ecological, evolutionary & environmental sciences

For a reference copy of the document with all sections, see nature.com/documents/nr-reporting-summary-flat.pdf

Life sciences study design

All studies must disclose on these points even when the disclosure is negative.

Sample size	Animal studies were designed so that each group/treatment had 4-6 animals to achieve statistical power according to our past experience as well as what has been reported in the literatures.
Data exclusions	No data was excluded in the study.
Replication	Specimens collected from the study included blood/sera, nasal wash samples, tissues, etc. For qPCR, each RNA sample was quantified in at least 2 replicates. Sara samples tested in ELISA and for neutralization were performed in duplicate in each assay and assays were repeated at least once.
Randomization	Male and female hamsters were used in the study and randomized in each group. Male BALB/c mice were housed in cages in groups-of-five, and were used without re-distribution (randomization) as the introduction of new cage-mates could result in fighting among mice.
Blinding	The study does not involve blinding.

Reporting for specific materials, systems and methods

We require information from authors about some types of materials, experimental systems and methods used in many studies. Here, indicate whether each material, system or method listed is relevant to your study. If you are not sure if a list item applies to your research, read the appropriate section before selecting a response.

Materials & experimental systems

n/a	Involved in the study	
☐	☒	Antibodies
☐	☒	Eukaryotic cell lines
☒	☐	Palaeontology and archaeology
☐	☒	Animals and other organisms
☒	☐	Human research participants
☒	☐	Clinical data
☒	☐	Dual use research of concern

Methods

n/a	Involved in the study	
☒	☐	ChIP-seq
☒	☐	Flow cytometry
☒	☐	MRI-based neuroimaging

Antibodies

Antibodies used	Western blotting: rabbit anti-SARS-CoV-2 spike polyclonal antibody (MyBioSource; cat. # MBS434243; lot T1218EL)<br>confocal microscopy: rabbit polyclonal anti-SARS-CoV-2 Spike antibody (GTX135356)<br>ELISAs: goat anti-mouse IgG, HRP conjugate (SouthernBiotech, cat # 1030-05, lot E2518-V099); goat anti-mouse IgM, HRP conjugate (SouthernBiotech, cat # 1020-05); goat anti-mouse IgG1, HRP conjugate (SouthernBiotech, cat # 1070-05, lot A5919-P199B); goat anti-mouse IgG2a, HRP conjugate (SouthernBiotech, cat # 1080-05, lot L2618-MN69); goat anti-mouse IgG2b, HRP conjugate (SouthernBiotech, cat # 1090-05, lot A2413-TK87F); goat anti-mouse IgG3, HRP conjugate (SouthernBiotech, cat # B2212-X539); and goat anti-hamster IgG, HRP conjugate (SouthernBiotech, cat # 6061-05, lot B3515-ZD09D).
Validation	Commercial antibodies were validated by the vendors.

Eukaryotic cell lines

Policy information about cell lines

Cell line source(s)	Vero, Vero E6 and DF-1 cells
Authentication	ATCC
Mycoplasma contamination	All cell lines have been tested and are free of mycoplasma.

Commonly misidentified lines (See ICLAC register)	*Name any commonly misidentified cell lines used in the study and provide a rationale for their use.*

Animals and other organisms

Policy information about studies involving animals; ARRIVE guidelines recommended for reporting animal research

Laboratory animals	BALB/c mice; Syrian Hamsters
Wild animals	*Provide details on animals observed in or captured in the field; report species, sex and age where possible. Describe how animals were caught and transported and what happened to captive animals after the study (if killed, explain why and describe method; if released, say where and when) OR state that the study did not involve wild animals.*
Field-collected samples	*For laboratory work with field-collected samples, describe all relevant parameters such as housing, maintenance, temperature, photoperiod and end-of-experiment protocol OR state that the study did not involve samples collected from the field.*
Ethics oversight	*Identify the organization(s) that approved or provided guidance on the study protocol, OR state that no ethical approval or guidance was required and explain why not.*

Note that full information on the approval of the study protocol must also be provided in the manuscript.
