# Supplementary material for: MVA vector expression of SARS-CoV-2 spike protein and protection of adult Syrian hamsters against SARS-CoV-2 challenge
Source: NPJ Vaccines. 2021 Dec 3;6:145. doi: 10.1038/s41541-021-00410-8 (PMC8642471; doi:10.1038/s41541-021-00410-8)
Supplement: Supplementary file 2 — Supplementary Information [file 41541_2021_410_MOESM2_ESM.pdf]

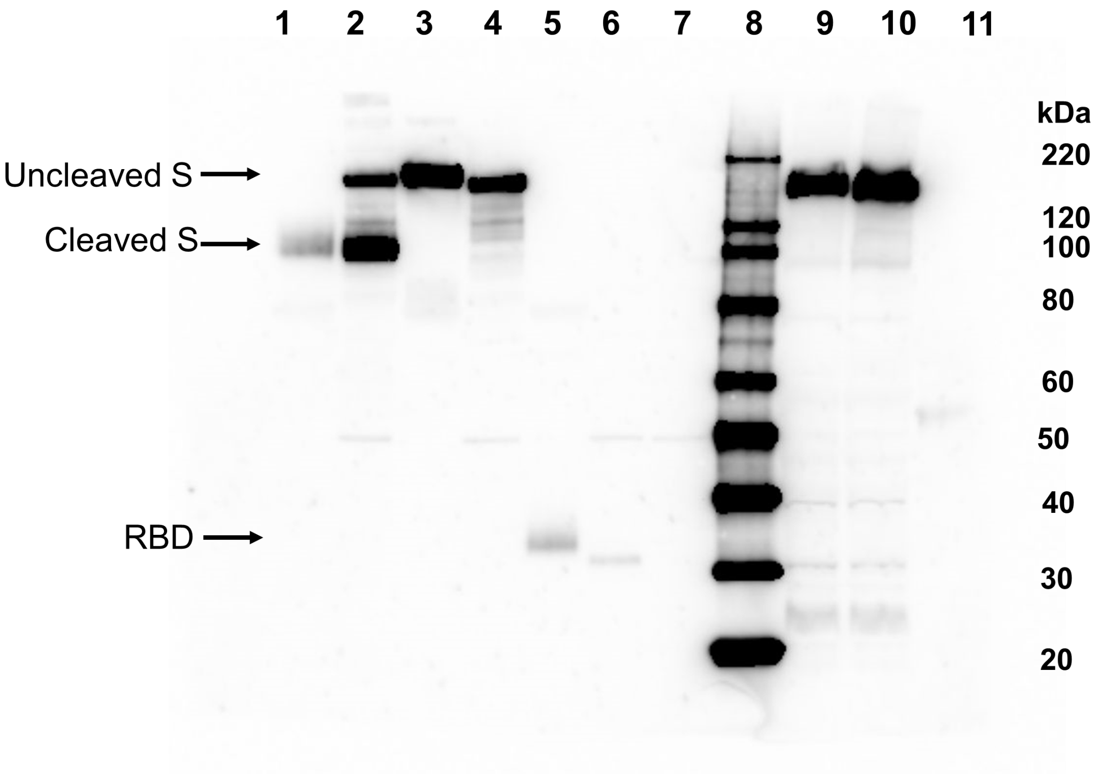

Supplemental Figure 1. Full, un-cropped image of the western blot of Figure 1b, including full molecular weight markers. Lane 11 is the rainbow protein molecular weight marker, which doesn't bind the secondary (HRP-conjugated) antibody, and cannot be detected by chemiluminescence. Lanes 9 &10 are not part of the current study.

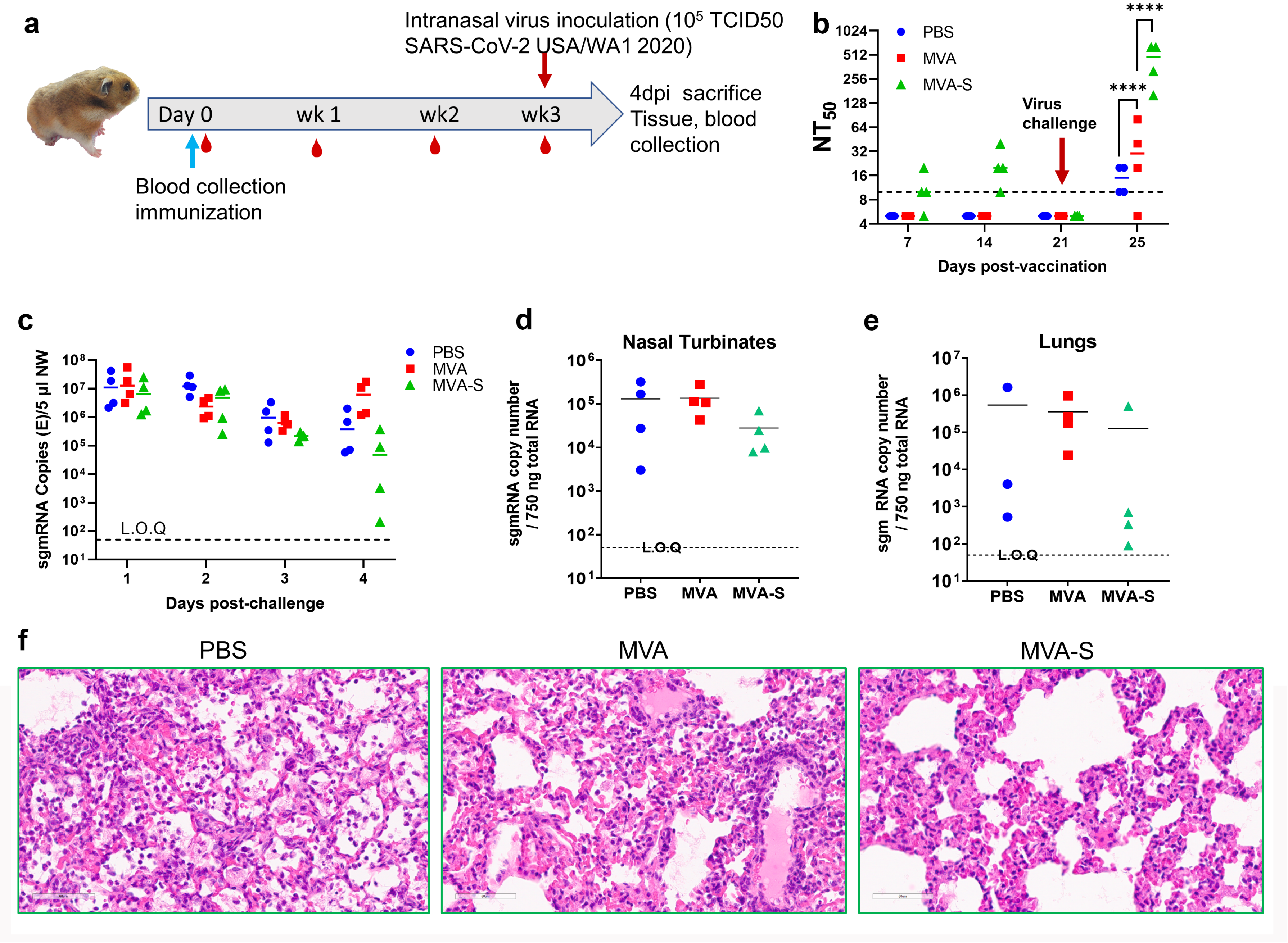

Supplemental Figure 2. Single vaccination with MVA-S modestly reduced viral loads in hamsters. (A) Adult Syrian hamsters (n=4 per group) were vaccinated with PBS, 108 pfu MVA, or 108 pfu MVA-S. Blood was collected weekly for three weeks. On day 21, hamsters were challenged with 105 TCID50 SARS-CoV-2 USA/WA1 and sacrificed on day 4 post-challenge. (B) Neutralizing antibody titers (nAB) were determined from the blood samples. Subgenomic viral mRNA (sgmRNA) levels were quantified in nasal wash samples collected at 1, 2,3, 4 day post-challenge (C), or from nasal turbinates (D) or lungs (E) collected at day 4 post-challenge. (F) Representative images of HE staining of lung tissues from lung tissues dissected at day 4 post-challenge. Hamsters vaccinated with only PBS or MVA showed around 20% or 15% consolidation areas, respectively, which are composed of type II pneumocyte hyperplasia and hypertrophy, small focal areas of atypical pneumocyte hyperplasia and hypertrophy, alveolar wall thickening, luminal infiltrates of foamy macrophages, lymphocytes, and neutrophils with lesser plasma cells. In and around these areas is mild to marked hemorrhage and moderate to marked proteinaceous fluid (edema). Hamsters vaccinated with MVA-S had very modest level of consolidation and signs of pneumonia.
